# Cochrane Cameroon: bringing Cochrane to francophone sub-Saharan Africa

**DOI:** 10.11604/pamj.2021.39.166.30508

**Published:** 2021-07-02

**Authors:** Lawrence Mbuagbaw, Guy Sadeu Wafeu, Tamara Kredo, Solange Durao, Joy Oliver, Charles Shey Wiysonge, Pierre Ongolo Zogo

**Affiliations:** 1 Centre for the Development of Best Practices in Health, Central Hospital, Yaoundé, Cameroon,; 2 Department of Health Research Methods, Evidence and Impact McMaster University, Hamilton, Canada,; 3 Biostatistics Unit, Father Sean O´Sullivan Research Centre, St Joseph´s Healthcare Hamilton, Hamilton, Ontario, Canada,; 4 Cochrane South Africa, South African Medical Research Council, Cape Town, Western Cape, South Africa,; 5 Division of Clinical Pharmacology, Faculty of Medicine and Health Sciences, Stellenbosch University, Cape Town, South Africa

**Keywords:** Cochrane, Cameroon, Africa, evidence-informed healthcare, francophone

## To the editors of the Pan African Medical Journal

June 30^th^ 2021 marks the launching of Cochrane Cameroon in Yaoundé, Cameroon. Cochrane Cameroon is the fourth geographical group of Cochrane in sub-Saharan Africa, following Cochrane South Africa (1997), Cochrane Nigeria (2006) and Cochrane Kenya (2021). All are part of the Cochrane Africa Network, formally established in 2017. Cochrane Cameroon is based in the Centre for Development of Best Practices in Health, at the Yaoundé Central Hospital in Cameroon, and is the base of the Francophone hub of Cochrane Africa [1]. The Francophone hub includes Benin, Burkina Faso, Cameroon, Congo, Democratic Republic of Congo, Ivory Coast, Madagascar, Mali and Senegal.

Cochrane is a not-for-profit international network aiming to have evidence at the heart of health decision-making world-wide. Cochrane does not accept commercial or conflicted funding which is vital for generating authoritative and reliable information, unconstrained by commercial and financial interests. Cochrane Cameroon responds to the needs to expand evidence-based decision-making and Cochrane activities to French speaking Africa [2] and to address broader health system deficiencies in sub-Saharan Africa [3, 4]. Cochrane Cameroon will provide leadership for the growing interest in evidence-based healthcare in Cameroon and the surrounding countries. Current efforts are limited by a lack of individual and institutional capacity to conduct and use systematic reviews [3]. Cochrane Cameroon has the unique opportunity to bridge the gap between the rest of Cochrane and French-speaking sub-Saharan Africa, as the sole bilingual (English/French) Cochrane geographical group in the region.

In collaboration with Cochrane Africa, Cochrane Cameroon will continue to support the production of high-quality systematic reviews, make relevant Cochrane evidence accessible (through translation of plain language summaries and evidence assessments), advocating for evidence (by raising awareness and stakeholder engagement) and contributing to the sustainability of the network (by supporting mentors and mentees). In recognition of the need to enhance health equity by involving stakeholders and end-users in priority setting [5], Cochrane Cameroon will build upon previous capacity-building efforts [6], and translation initiatives [7], to ensure that French-speaking people in sub-Saharan Africa are engaged in the evidence generation process.

**Conclusion:** this letter is an open invitation to researchers, health care providers, journalists, policymakers, and consumers in the region to visit our website and engage meaningfully in the production and use of high-quality locally relevant and accessible health evidence.
